# Filtration Behaviour and Fouling Mechanisms of Polysaccharides 

**DOI:** 10.3390/membranes4030319

**Published:** 2014-07-08

**Authors:** Sondus Jamal, Sheng Chang, Hongde Zhou

**Affiliations:** School of Engineering, University of Guelph, Guelph, ON N1G 2W1, Canada; E-Mails: sjamal@uoguelph.ca (S.J.); hzhou@uoguelph.ca (H.Z.)

**Keywords:** membrane fouling, soluble microbial product (SMP), MBR, concentration polarization, polysaccharides, alginate

## Abstract

This study investigated filtration behaviors of polysaccharides solutions, both alone and in mixture with proteins, in the short-time constant flux filtration with the focus on factors affecting the transmembrane pressure (TMP) increase rate, the irreversible filtration resistance, and the membrane rejection behavior. The results showed that the TMP increase rates in the short-time constant flux filtration of alginate solutions were significantly affected by the calcium addition, alginate concentration, and flux. Although the addition of calcium resulted in a decrease in the TMP increase rate, it was found that the irreversible fouling developed during the filtration increased with the calcium addition, implying that the double-sided effect of calcium on membrane filtration and that the TMP increase rate observed in the filtration does not always reflect the irreversible membrane fouling development. It was also found that for the filtration of solutions containing mixed alginate and BSA, alginate exerted a dominant effect on the TMP increase rate and the membrane exhibited a reduced rejection to both alginate and BSA molecules compared to that in the filtration of the pure alginate or BSA.

## 1. Introduction

The applications of membrane bioreactors (MBR) technology in municipal and industrial wastewater treatment have been expanded in recent years. However, membrane fouling still remains a critical factor affecting the capital and operational costs of MBRs. In the case of wastewater treatment, mixed liquor contains flocculated bioactive suspended solids, colloidal and soluble microbial products (SMP) as well as dissolved inorganic substances. Current fouling control strategies such as air scouring and flux regulation are proven to be effective to control cake formation and concentration polarization in MBRs. However, SMPs that are mainly composed of polysaccharides and proteins, are regarded as a major foulant affecting long-term membrane fouling in MBRs under air scouring and controlled flux condition [1,2,3]. Thus, an examination of the fouling mechanism of polysaccharides and proteins is important for controlling membrane fouling in MBRs. 

Alginate has been used as a model polysaccharide to study the filtration behaviour and fouling mechanisms of SMPs in many studies [4,5,6,7,8,9,10]*.* Van den Brink *et al.* [11] found that calcium addition reduced the TMP increase rate but increased the irreversibility of the fouling. Listiarini *et al.* [12] suggested that cake formation was the main resistance mechanism in the filtration of alginate solution and reported that the cake layer formed by the alginate exhibited an incompressible behaviour in both the presence and absence of calcium. Nataraj *et al.* [5] also indicated that the cake filtration model fit the entire range of their ultrafiltration data for alginate solutions at a concentration of 20 ppm. In contrast, Katsoufidou *et al.* [13] reported that the internal pore constriction at the beginning of filtration resulted in a rapid irreversible fouling stage in the filtration of alginate without added calcium, but with calcium, cake formation was dominant throughout the filtration and the fouling reversibility was increased. In a later study, Katsoufidou *et al.* [8] found that the fouling behaviour of sodium alginate was dominant in the filtration of solution containing sodium alginate (SA) and humic acid (HA) such that the behaviour of HA/SA mixtures was quite similar to that of alginate alone even when SA was present in a small proportion. It was also reported that the membrane exhibited a higher rejection to the HA and SA when they were mixed in the solution [8]. Mo *et al.* [10] found that the effect of calcium addition on alginate fouling depended on the calcium concentration with a positive effect observed at the concentration above a critical value. Thus, most of the previous studies showed that the addition of calcium reduced the filtration resistance caused by the alginate, but increased its irreversibility in the filtration with intermittent permeation [11], or with backwashing [8], or during a relatively long filtration period [12,13].

In this study, the focus is on the filtration behavior of alginate solution in the short-time constant flux filtration mode. The duration of the short-time filtration can be arbitrarily defined between 15 min and one hour. In this study, a one-hour filtration duration was used. The interest in the short-time filtration mode are because this filtration mode has been widely used in the critical flux and sludge filterability tests. In these tests, the TMP increase rate has been used as the main criteria or indicator to determine the operation flux, sludge filterability, and the fouling potential of solutions [14,15]. The aim of this paper is to analyze the typical TMP-time profiles observed in the short-time constant flux filtration of alginate solutions, and examine the effects of calcium addition, change of flux, and the presence of protein on the TMP increased rate and the irreversible fouling development. Another parameter under investigation is the membrane rejection behavior to the protein and polysaccharides in the short-time constant flux filtration. The results presented in this paper can develop improved understanding of the factors affecting polysaccharide fouling and the membrane methods to assess the membrane-fouling propensity of polysaccharides.

## 2. Materials and Methods

**Figure 1:**
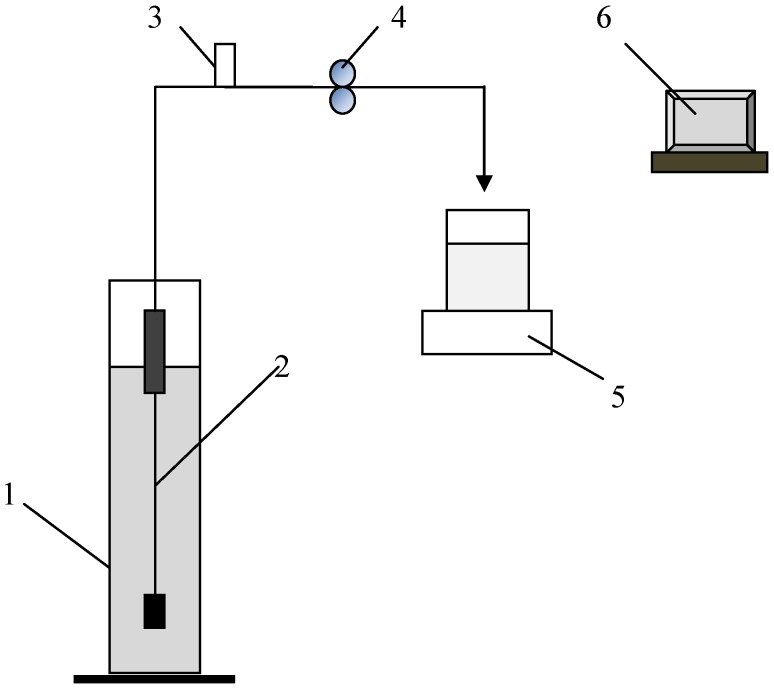
Schematic of the experimental setup.

The single fiber experimental set-up shown in Figure 1 was used for the filtration experiments. The filtration system consisted of a feed tank (1), a single hollow fiber module (2), a pressure transducer with a pressure range −14.5 to 15 psi (Cole Parmer, 68075-32) (3), a peristaltic pump (Minipuls 3, Gilson) (4), a balance (CPA6235, Sartorius) for the permeate flow measurement (5), and a computer data logging system (6). The single hollow fiber membrane module used was made from PVDF hollow fibres with a membrane pore size around 0.04 μm (GE Water Process Technologies, Canada). The fiber length was 22 cm with a 0.9 mm ID and 1.8 mm OD, providing a total filtration area of 14 cm^2^. The filtration experiments were carried out in constant flux mode. Fresh hollow fiber membranes were used for each filtration test and the membrane resistance was determined by clean water filtration. The irreversible membrane fouling (ΔR_m_ %) developed during the filtration was assessed by comparing the membrane resistance before and after the filtration. 

The chemicals used in this study, including sodium alginate (SA), bovine serum albumin (BSA), sodium bicarbonate (NaHCO_3_), and calcium chloride (CaCl_2_), were produced by Acros Organics (Fisher Scientific). A 10 g/L alginate solution was made by dissolving the alginate into deionised water with mechanical stirring for around 3 h to ensure the fully dissolution of the alginate. It was then stored in the refrigerator as the stock solution that was diluted to 1 g/L for the zeta potential measurement and to the desired concentration for the filtration experiments (10 and 20 mg/L). Sodium bicarbonate of 2 mM was used as a buffer of the alginate solutions used in the filtration. CaCl_2_ was used to adjust the Ca^2+^ concentration of 0, 6.25, 12.5, 18.75, 25, 50, 100, 222.5 mg/L for testing effect of calcium on alginate filtration behavior. For the protein mixture experiments, a 5 g/L BSA stock solution was made by dissolving the BSA into deionised water and then stored in the refrigerator, and used for making the BSA solutions of different concentrations (0, 2.5, 5, 10, 20 mg/L). 

The zeta potential of the alginate was measured using a Zetasizer Nano Z instrument (Malvern). Spectrophotometric and colorimetric methods have been used for protein and carbohydrate analysis [16,17]. The protein rejection analysis was carried out using a modified BCA protein assay method for lower concentration samples. The Pierce BCA test kit (cat. No.: 23225, 23227) was used and the Enhanced Protocol was followed with BSA as a standard. The coverage range for the protein calibration curve was 0–250 ppm with 8 measured calibration points. The alginate rejection was carried out in accordance with a modified version of the method of Dubois *et al.* [16] for lower concentration samples with glucose solution used as a standard. The coverage range of the carbohydrate calibration curve was 0–70 ppm with 8 measured points. The HACH DR 4000 spectrophotometer was used for the absorbance measurement at 562 nm for protein and at 490 nm for carbohydrate. 

## 3. Results and Discussion

### 3.1. Zeta Potential of Alginate Molecules

Zeta potential reflects the charge density of colloids, which dominates the inter-molecular interactions through long-range repulsive forces. It is thus usually used to assess the aggregation potential of colloids and particulates in water and wastewater. For the membrane filtration, measurement of zeta potential will aid in prediction of long-term colloidal stability of the macromolecules in the concentration polarization (CP) layer formed on the membrane surface. 

**Figure 2:**
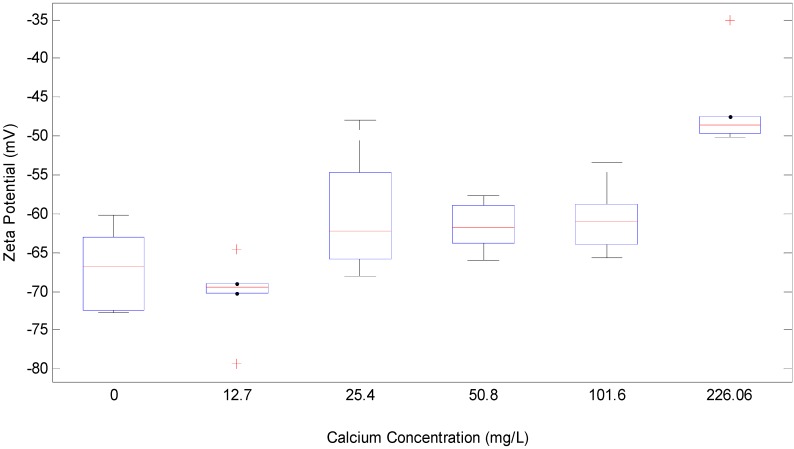
Box plot for change in Zeta potential (mV) with calcium concentration (mg/L) in 1 g/L SA and 2 mM NaHCO_3_ buffer.

Figure 2 is the box plot for the effect of calcium concentration on the zeta potential of the alginate tested. The measurement showed that the zeta potential of the tested alginate was reduced from −70.3 mV to −46 mV when Ca^2+^ concentration added increased from 0 to 222.5 mg/L. Significant gelling was observed when further increasing the calcium concentration beyond this range. 

The zeta potential reduction caused by calcium addition could be attributed to the aggregation of alginate molecules. It is well known that Ca^2+^ can selectively bond to guluronic acid (**G**) residues of alginate to form a crosslink between alginate molecules, which can result in the formation of gel aggregates or lumps in a dilute alginate solution [18,19]. Thus, calcium addition can affect the filtration behavior of the alginate through changing the molecular configurations, size, and surface charge of alginate molecules. 

### 3.2. Short-Time Filtration Behavior of Alginate

**Figure 3:**
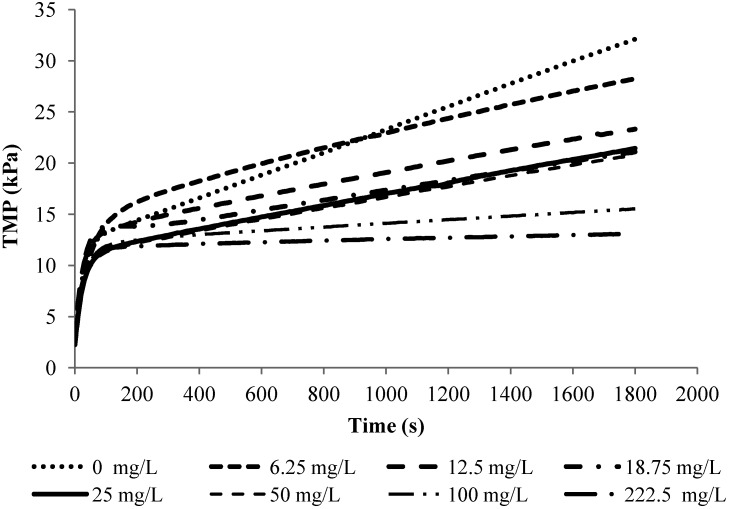
TMP profiles for experiments with 10 mg/L SA and 2 mM NaHCO_3_ at flux 40 LMH.

Figure 3 shows the TMP-time profiles obtained in the filtration of 10 mg/L alginate solutions with different calcium concentrations at a flux of 40 L/m^2^/h (LMH). From this figure, it can be seen that the TMP profiles for the filtration of alginate solutions under constant flux condition can be characterized by a non-linear initial TMP rise followed by a linear TMP increase. Such a profile was observed in the filtration experiments with alginate solutions of different solution chemistry and concentrations. In fact, such a non-linear/linear TMP profile has also been a typical TMP profile observed in the critical flux tests with mixed liquor in MBRs [15,20]. A general practice in the critical flux and sludge filterability test is to characterize the sludge fouling propensity and filterability based on the linear TMP increase rate.

#### 3.2.1. Factors Affecting the Linear TMP Increase Rate

The linear TMP increase rate depends on the resistance and the growth rate of the CP layer. The experiments conducted in this study showed that the linear TMP increase rate of alginate solution could be affected by calcium addition, flux, and the presence of protein. 

**Figure 4:**
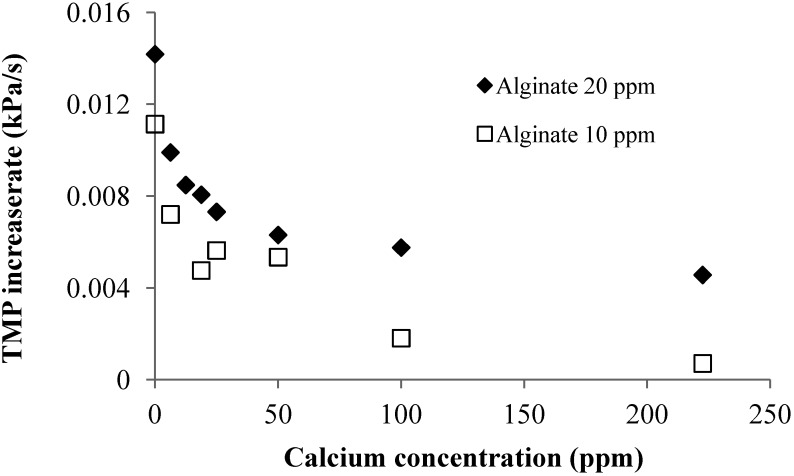
Effect of calcium addition on TMP increase rate at different SA concentrations (flux 40 LMH; 2 mM NaHCO_3_ solution).

Effect of Calcium Addition: Figure 4 shows the effect of calcium addition on the linear TMP increase rate in the filtration of 10 and 20 mg/L alginate solutions. The experiments showed that the TMP linear increase rate decreased with increase in the calcium concentration. The effect of the calcium addition was more pronounced at the low calcium concentration range for both 10 and 20 mg/L alginate solution. As discussed, calcium addition can result in the formation of alginate gel aggregates and reduction in molecular surface charge. The later will reduce the repulsive force between the molecules, which can lead to a more tightly packed CP layer. Thus, the reduced TMP increase rate with the calcium addition should be attributed to the formation of gel aggregates, which will increase the size of alginate, resulting in a reduced resistance of the CP layer. 

**Figure 5:**
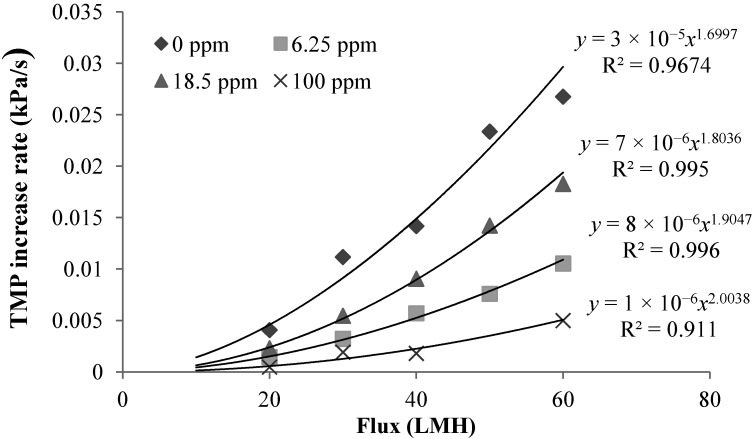
Effect of flux on TMP rate for filtration with different calcium concentrations (calcium concentrations: 0 ppm, 6.25 ppm, 18.5 ppm and 100 ppm) at 10 ppm sodium alginate concentration and 2 mM sodium bicarbonate concentration.

Effect of Operation Flux: Figure 5 shows the effect of the operation flux on the TMP increase rate. It is evident that the linear TMP increase rate increases with the flux. Fitting the experimentally determined TMP increase rate-flux relations with power function trend lines showed that the dTMP/d*t* obtained in the filtration of alginate solutions with different calcium concentrations can be affected by the flux to different indexes, indicating that the properties of the CP layers varies with the concentration of added calcium (Figure 5).

Assuming that the linear TMP increase rate is caused by the growth of CP layer at a constant concentration, the linear TMP increase rate under the constant flux filtration condition can be linked to the specific resistance of CP layer by [21]:

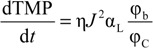
(1)
where η is the water viscosity; J is the flux; α_L_ is the thickness-based specific resistance; ϕ_b_ is the solid volume fraction of the bulk solution; and ϕ_C_ is the solid fraction in the CP layer. 

Then, the ratio of the specific resistance to the solid volume fraction of the CP layer can be expressed as:

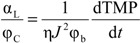
(2)
The parameter α_L_/ϕ_C_ reflects the filtration resistance caused by solids, measured in volume, in the unit volume of CP layer. Based on the experimentally determined flux dTMP/dt relationshp, the index for the α_L_/ϕ_C_ can be determined using Equation (2). Table 1 summarized the index of dTMP/d*t* and α_L_/ϕ_C_ for the filtration of alginate solutions of different calcium concentrations. A positive index for the α_L_/ϕ_C_ indicates a compressible CP layer because the α_L_/ϕ_C_ increases with the flux or the hydraulic drag, while the CP layer is incompressible for an index close to zero. From Table 1, it can be seen that the CP layers for the filtration of alginate solutions with calcium concentration of 18.5 and 100 mg/L exhibited an index close to zero, indicating an incompressible behavior of the CP layers. However, it is interesting to note that the α_L_/ϕ_C_ in the flux range of 20 to 60 LMH showed a negative index in the filtration of the alginate solution without calcium addition and at the calcium concentration 6.25 mg/L. The negative index suggests that the resistance caused by the solids per unit volume of CP concentrate was reduced with increase in the flux. Such a trend is difficult to explain by the conventional filtration theory without considering the effect of the molecular properties of alginate. Our recent research revealed that the alginate used in this study had a molecular weight (*M_w_*) of 186 kDa and a Mark-Houwink α parameter of 1.01 by using a size exclusion chromatography (SEC) equipped with the light scattering detectors and the viscometer [22]. The α parameter above 0.8 indicates that the alginate is a semi-flexible polymer with a molecular configuration close to a rod-shaped polymers. A reduced α_L_/ϕ_C_ value with the increased flux may suggest that the increased flux resulted in an increased packing randomness or lower packing density of the alginate molecules in the concentration polarization layer. On the other hand, it is known that concentrated alginate solutions can exhibit Non-Newtonian shear-thinning behavior, which results in a reduced viscosity with increase in the shear rate [23]. Thus, the resistance behavior of the concentration formed by alginate under the low flux condition, where the retained alginate has not been fully compressed, may be different from those described by the conventional filtration theory due to the effect of the molecular properties of alginate. Thus, the change in the index of α_L_/ϕ_C_ reflects the role of the molecule configuration of polysaccharide molecules in determination of resistance characteristics of CP layer formed in filtration of polysaccharide solution. 

**Table 1:** Flux effect power index for dTMP/d*t* and α_L_/ϕ_C._

Calcium Concentration (mg/L)	*a* for dTMP/d*t* ∝ *J*^a^	*b* for α_L_/ϕ_C_ ∝ *J*^b^
0	1.70	−0.3
6.25	1.80	−0.20
18.5	1.97	−0.03
100	2.00	−0.00

**Figure 6:**
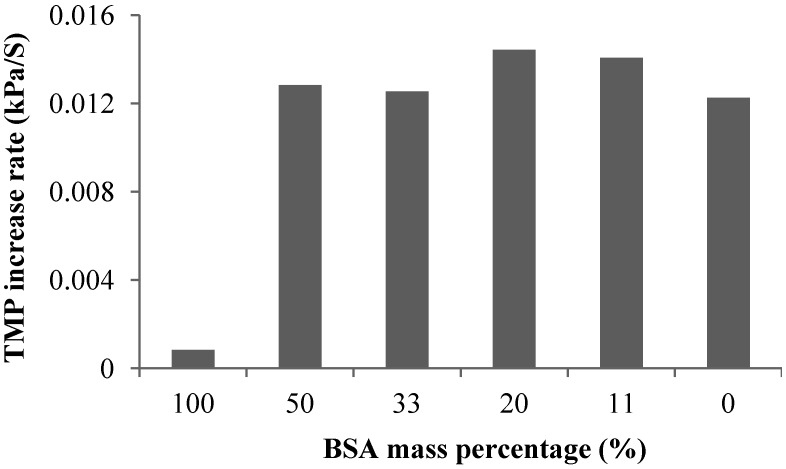
Effect of protein addition on TMP rate.

Effect of Protein Presence: The main components of SMPs are protein and polysaccharides. To investigate the effect of protein presence on the filtration behavior of alginate solutions, filtration experiments were conducted using alginate solutions with different BSA concentrations. As shown in Figure 6, the experiments showed that the linear TMP increase rate caused by BSA was significantly lower than that by alginate. For the solution containing the mixture of BSA and alginate, the alginate was found to exert a dominant effect on the linear TMP increase rate, while the effect of BSA on the TMP increase rate in the short-time constant flux filtration was found insignificant. The insignificant effect of the BSA presence on the TMP increase rate is understandable given the large difference in the molecular weight and configuration between alginate and BSA molecules, which may suggest that it is the alginate that forms the structure of the CP layer in the filtration of the solution of protein and alginate mixture.

### 3.3. Factors Affecting Irreversible Membrane Resistance

**Figure 7:**
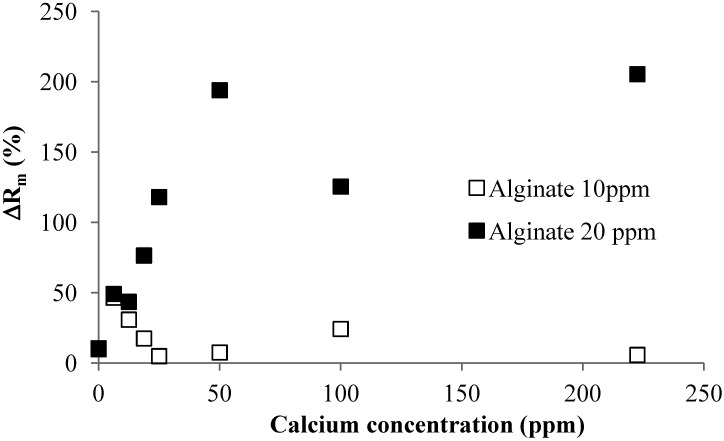
Effect of calcium addition on irreversible resistance (flux 40 LMH; 2 mM NaHCO_3_ solution).

Irreversible fouling is caused by molecules or colloids firmly attached to the membrane surface and pores, which results in the changes in membrane permeability. In this study, irreversible resistance is characterized by comparing the membrane resistance before and after the filtration by using clean water filtration testing. Figure 7 shows the membrane resistance change after 60 min filtration of alginate solutions of different calcium concentrations at flux 40 LMH. The experiments showed that the addition of calcium exerted an evident effect on the irreversible fouling development in the filtration of the 20 ppm alginate solutions, where the irreversible resistance increased with calcium concentration at the low calcium concentration range but leveled off at the relatively high calcium concentration. However, it was noticed that the effect of calcium on the irreversible resistance was less profound with the 10 ppm alginate solutions, although a negative effect of calcium on the irreversible resistance was also observed. The effect of the calcium on the irreversible resistance in the low calcium concentration range (6.25–25 mg/L) indicates the role of Ca^2+^ in affecting the membrane-alginate interactions. The calcium ions increased the attachment of the alginate molecules on the membrane surface through reducing the negative charge of the alginate molecules and/or forming positive charged contact points along the alginate molecular chains that will “fasten” the alginate molecules on the negatively charged membrane surface. However, the increase in the calcium concentration could result in the formation of alginate aggregates which can form a more reversible cake layer, probably due to the largely reduced specific contact surface area with the large aggregates formed under high calcium concentration.

**Figure 8:**
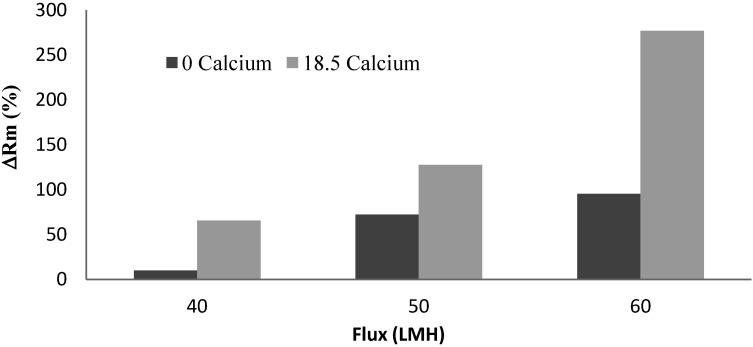
Effect of operational flux on irreversible resistance.

Figure 8 shows the effect of the flux on the irreversible resistance in the flux range 40 to 60 LMH for the short-time constant flux filtration of the alginate solution with and without calcium addition. In these experiments, the total filtrate volume was maintained the same by adjusting the filtration time. As expected, the increased flux resulted in more serious membrane fouling for the same solution condition. 

**Figure 9:**
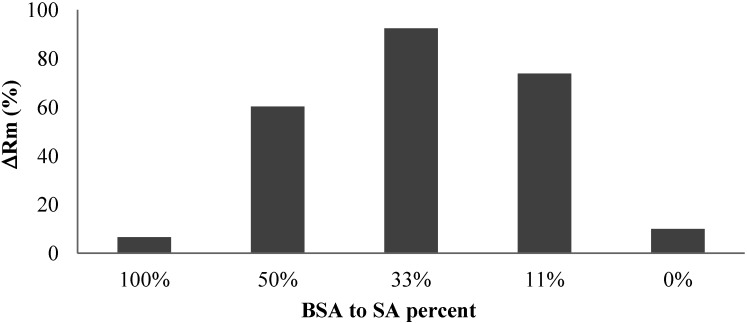
Effect of protein addition on irreversible resistance.

Figure 9 shows the effect of protein addition on the irreversible resistance. It was found that the addition of protein significantly increased the development of the irreversible resistance although both pure BSA and alginate solution caused insignificant irreversible fouling after 60 min filtration. The experiments also showed that the mass ratio of BSA to alginate affecting the irreversible fouling development with the highest irreversible fouling observed in the filtration of solution with a BSA to alginate ratio around 33%.

**Figure 10:**
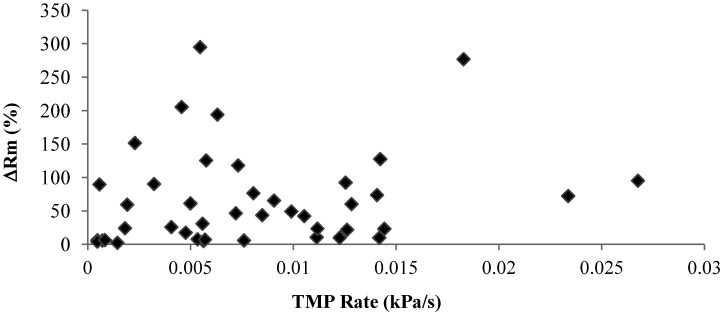
Relationship between irreversible resistances and TMP rate (kPa/s).

Irreversible fouling is a key factor affecting the long-term stable operation of membrane filtration. Thus, determining the irreversible fouling potential of mixed liquor or other filtration solutions is of critical importance for the membrane filtration operation and design. Although the TMP increase rate is commonly used as a fouling potential indicator, this study shows that the TMP increased rate observed in the short constant flux filtration may not be a reliable indicator to predict the membrane fouling behavior of polysaccharide. Figure 10 shows the irreversible resistance developed during the filtration against the corresponding TMP increase rate, which shows that there is no direct relationship between the TMP increase rate and the irreversible fouling development, which suggested that it is necessary to measure both the TMP increase rate and the membrane resistance before and after the filtration to determine the fouling potential of the filtration solutions. 

### 3.4. Membrane Rejection to Alginate and BSA

**Figure 11:**
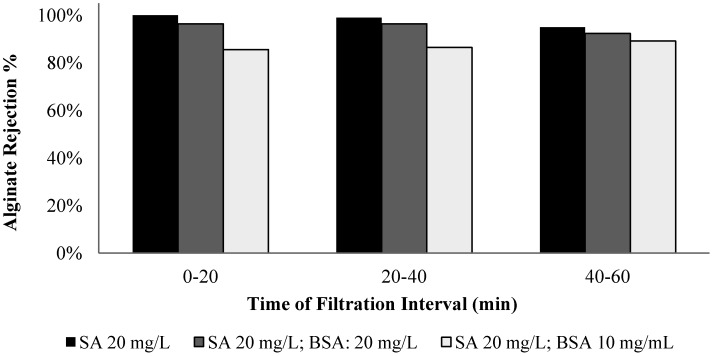
Alginate rejection for filtration of solutions with different ratios of BSA to SA.

**Figure 12:**
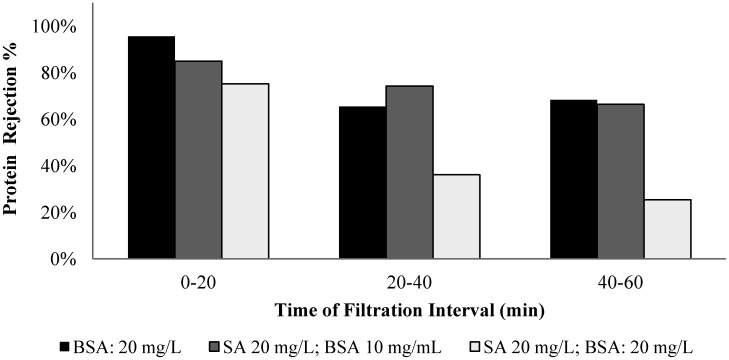
Protein rejection for filtration of solutions with different ratios of BSA to SA.

The membrane rejection behavior to alginate and BSA was assessed by conducting filtration experiments with solutions of different alginate/BSA compositions, including solutions with alginate (mg/L)/BSA (mg/L) ratios of 20/0, 20/10, 20/20, and 0/20. Samples were taken in the filtration time periods of 0 to 20, 20 to 40, and 40 to 60 min for the carbohydrates and protein analysis. Figure 11 and Figure 12 show the alginate and BSA rejection during the different sampling periods for the solutions tested. 

The membrane exhibited an average 98% rejection to alginate in the filtration of pure alginate solution and the highest rejection occurred in the 0 to 20 min period. It is interesting to note that the membrane rejection to alginate was lower for the mixed alginate/BSA solution, particularly for the solution with 20 mg/L alginate and 10 mg/L BSA. For the pure BSA solution, the membrane exhibited an average rejection of 76% over the one-hour filtration period with an evident low rejection in the time periods of 20 to 40 and 40 to 60 min. The presence of the alginate also resulted in a reduction in the protein rejection and the largest reduction occurred with the solution containing 50% protein and alginate.

The transmission of the alginate or protein molecules from the feed to the permeate side depends on the rejection characteristics of the membrane and the CP layer. The reduced rejection with the filtration time suggests that the effect of concentration polarization on the observed membrane retention. But it is also worth noting that the experimental results obtained in this study showed that the CP layer formed by the mixture of alginate and BSA exhibited a lower rejection to both alginate and BSA molecules. As the water permeates through the concentration polarization layer, the concentration of the polysaccharides or proteins in the permeate flow can be assumed in equilibrium with that of polysaccharides or proteins in the concentration polarization layer. An increased polysaccharide or protein concentration in the permeate flow (or a reduced membrane rejection) may suggest an increase in the chemical potential of the polysaccharides or protein in the concentration polarization layer, which could result from weakened intermolecular interactions caused by the mixing of the protein and alginate.

## 4. Conclusions

The short-time constant flux filtration mode is widely used in the critical flux test and sludge filterability measurement. This study showed that for the short-time constant flux filtration of alginate solutions, the TMP time profiles can be characterized by a non-linear TMP rise, followed by a linear TMP increase. The linear TMP increase rate can be significantly affected by the presence of calcium ions and the flux condition. For the filtration of the mixed alginate and BSA, the alginate exerted a dominant effect on the TMP increase rate. It was found that the irreversible fouling developed through the filtration period increased with the addition of calcium, and that the TMP increase rate exhibited in the short-time constant flux filtration did not always reflect the development of the irreversible fouling during the filtration period. For membrane rejection to the alginate and BSA, it was found that the CP layers formed by the mixed alginate and BSA exhibited a lower rejection to both alginate and BSA molecules compared to the CP layers formed by pure alginate or BSA molecules. 
